# Evaluation of National Academies of Sciences, Engineering, and Medicine (NASEM, 2021) feed evaluation model on predictions of milk protein yield on Québec commercial dairy farms

**DOI:** 10.3168/jdsc.2024-0549

**Published:** 2024-04-20

**Authors:** S. Binggeli, H. Lapierre, R. Martineau, D.R. Ouellet, E. Charbonneau, D. Pellerin

**Affiliations:** 1 Département des sciences animales, Université Laval, Québec, QC, Canada G1V 0A6; 2 Sherbrooke Research and Development Centre, Agriculture and Agri-Food Canada, Sherbrooke, QC, Canada, J1M 0C8

## Abstract

A recent study assessed the ability of 4 feed evaluation models to predict milk protein yield (MPY) in a commercial context, with data of 541 cows from 23 dairy herds in the province of Québec, Canada. However, the recently published Nutrient Requirements of Dairy Cattle from the National Academies of Sciences, Engineering, and Medicine (NASEM, 2021) was not released at that time. Thus, the current study evaluated NASEM using the same dataset. To be consistent with the previous study, predicted DMI was used. Therefore, MPY was predicted using the 2 estimations of DMI proposed by NASEM: one based on animal characteristics only (DMI_Ao_) and one also including ration characteristics (DMI_A&R_). For each type of DMI estimates, 2 MPY predictions were made, using (1) the multivariate equation directly published in NASEM and (2) a variable efficiency of utilization of MP predicted using inputs and outputs from NASEM, published a posteriori. With the 2 approaches, multivariate and variable efficiency, the DMI_A&R_ yielded the best MPY predictions. The multivariate equation showed a regression bias between observed and predicted MPY with both DMI estimations. The estimated variable efficiency allowed for MPY predictions without mean and regression biases. With DMI_A&R_, concordance correlation coefficients (CCC) were 0.72 and 0.78 for MPY predicted using the multivariate and variable efficiency equations, respectively. In comparison, DMI_Ao_ CCC were 0.60 and 0.71, respectively. In conclusion, on commercial farms, where dairy rations are usually optimized for a group of cows, estimates of DMI based on animal and rations characteristics yielded the best MPY predictions. The multivariate equation from NASEM predicted MPY with a regression bias, whereas the variable efficiency of utilization of MP based on MP and energy supplies resulted in no bias in MPY predictions.

## Highlights

• The 2021 NASEM model proposed a multivariate equation to predict milk protein yield.
• Milk protein yield can be predicted from an efficiency of utilization of protein.
• The variable efficiency of utilization of protein yielded the best predictions.
• NASEM proposed 2 equations to predict dry matter intake based on cow and/or diet.
• Dry matter intake equation 2 yielded the best predictions of milk protein yield.

## Graphical Abstract

**Figure:**
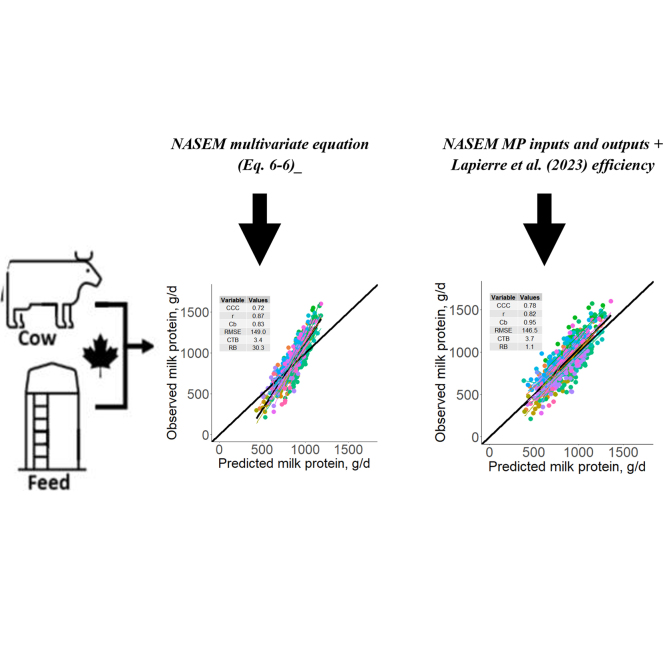
**Summary:** The NASEM (2021) model was evaluated for its ability to predict milk protein yield using data from commercial Canadian dairy farms. The multivariate equation proposed by NASEM showed a regression bias between observed and predicted yields. A second set of predictions was calculated based on the predicted variable efficiency of utilization of metabolizable protein, derived from NASEM inputs and outputs based on protein and energy supplies and days in milk. It yielded predictions without mean and regression biases, indicating its promising usage for milk protein yield prediction in commercial herds.

A recent study compared 2 American (National Research Council, 2001; Cornell Net Carbohydrate and Protein System, v 6.5.5, van Amburgh et al., 2015) and 2 European (NorFor, Volden, 2011; INRA, 2018) feed evaluation models (**FEM**) on their ability to predict milk protein yield (**MPY**) on commercial dairy farms (Binggeli et al., 2022). In that article, the eighth revised edition of the Nutrient Requirement of Dairy Cattle by the National Academies of Sciences, Engineering and Medicine (**NASEM**, 2021) could not be evaluated because it had not been released yet. The NASEM (2021) revisited protein, AA, and energy supplies and developed a multivariate equation to predict MPY based on dietary supplies and BW. The coefficients of the multivariate equation are adjusted based on the rolling herd average (**RHA**) of the herd. Furthermore, using the inputs and outputs of NASEM, Lapierre et al. (2023) developed an equation to predict the efficiency of utilization of MP based on the ratio of MP supply minus endogenous urinary MP on digestible energy intake and DIM, which could be used to predict MPY. Therefore, the objective of this study was to evaluate the ability of the NASEM multivariate equation and the estimated variable efficiency of utilization of MP to predict MPY, using the dataset of commercial dairy used in Binggeli et al. (2022).

For model evaluation, the database used was the same as described by Binggeli et al. (2022, 2023) and Fadul-Pacheco et al. (2017). Diet and production data were selected only if all individual feed ingredients were known, and thus the diets did not contain any commercial concentrate mixes, except for minerals. Shortly, data were collected from 23 commercial dairy farms in Québec, Canada, and resulted in 541 cow BW, observed MPY, and their respective diets. On milk test day, milk yield was recorded, feed and diet information were collected, and milk samples were analyzed using near-infrared spectroscopy (Foss MilkoScan FT 6000) by Lactanet (Sainte-Anne-de-Bellevue, QC, Canada). Concentrate feeds and forages were analyzed using wet chemistry (SGS Agrifood Laboratories, Guelph, ON, Canada) and infrared methods (Lactanet), respectively. Laboratory analyses included DM, NDF, ADF, NDIN, ADIN, lignin, ether extract, starch, ash, and ash. All feed ingredients were analyzed for these main components. For missing analyses, such as protein degradability coefficients of AA and fatty acid concentration, the table values from the NASEM feed table for each feed ingredient were used. The digestibility of NDF was estimated using the lignin-based prediction equation from NASEM (2021; Equation 3–3a), whereas reference feed table was used for starch digestibility. The RHA, used by NASEM to adjust the coefficients of the multivariate equation, was determined from the 305-milk protein production available with the DHI data and was calculated for each cow individually. When missing (n = 13), the value of 280 kg of protein/305 d was used as suggested by NASEM. The RHA averaged 304 ± 57 kg/305 d (Table 1).

**Table 1:** Descriptive statistics of diets, cows, and farms used for analysis

Item	Mean	SD	Minimum	Maximum	n
Diet					
ADF, % of DM	23.6	3.2	17.1	35.9	541
NDF, % of DM	38.8	4.1	32.0	54.1	541
CP, % of DM	15.5	1.1	11.6	17.9	541
Forage					
fNDF, % of forage DM	51.3	7.6	36.4	66.8	58^1^
fNDF digestibility,^2^ % of fNDF	53.4	5.8	42.5	67.2	58
CP, % of forage DM	14.1	4.5	3.6	24.4	58
Cow					
Parity	2.4	1.5	1	8	541
BW,^3^ kg	672	57.7	535	841	541
DIM	202	116	22	684	541
Milk yield, kg/d	29.1	9.5	5.9	63.8	541
Milk protein concentration, %	3.31	0.39	2.51	4.67	541
Milk protein yield, g/d	894	248	211	1,601	541
Rolling herd average, kg/305 d	304	57	151	479	541
Herd average^4^					
Lactating cows per herd, n	44.8	16.9	23	98	23^1^
Parity	2.4	0.28	1.8	3.0	23
BW, kg	676	20	632	705	23
DIM	184	27	129	231	23
Milk yield, kg/d	30.3	3.2	23.5	36.2	23
Milk protein concentration, %	3.35	0.13	3.05	3.58	23
Milk protein yield, g/d	928	107	709	1,170	23
Rolling herd average, kg/305 d	303	28	238	345	23

1 Represent the individual number of forage and herd, respectively.

2 48-h in vitro incubation, calculated following the equation 20–110 from NASEM (2021).

3 Using thoracic circumference (Yan et al., 2009).

4 Some cows from some herds were excluded from the analysis.

Diet evaluations were made using R-based code functions distributed with the NASEM (2021) program V8 R2021.12.29, downloaded from https://www.nap.edu/catalog/25806/nutrient-requirements-of-dairy-cattle-eighth-revised-edition, January 2022. All diet evaluations were processed directly on R (R Core Team, 2022).

Two approaches to predict MPY were assessed in the current study. First, MPY was predicted using the multivariate equation proposed by NASEM (2021; Equation 6–6 in chapter 6 of the book) and herein is referred to as **MPY_multi**.

[1] $$\begin{array}{l} MPY\_multi=[-97.0+1.68\times His+0.885\times Ile+0.466\\ \times Leu+1.15\times Lys+1.84\times Met+0.077\times OAA-0.00215\\ \times EAA^{2}+10.8\times DEInp-4.6\times \left(dNDF-17.06\right)-0.42\\ \times \left(BW-612\right)], \end{array}$$

where *His* = His supply (g/d); *Ile* = Ile supply (g/d); *Leu* = Leu supply (g/d); *Lys* = Lys supply (g/d); *Met* = Met supply (g/d); *OAA* = supply of NEAA + Arg + Phe + Thr + Trp + Val (g/d); *EAA* = sum of the supply of 5 EAA squared; His^2^ + Ile^2^ + Leu^2^ + Lys^2^ + Met^2^ (g^2^/d^2^); *DEInp* = nonprotein digestible energy intake (Mcal/d); *dNDF* = digestible NDF (% DM); and BW (kg).

Second, MPY was predicted using the equation based on a variable efficiency of utilization of MP (*Eff*; %) as suggested by Lapierre et al. (2023):

[2] $$Eff=\;176-21.2\times Ratio+0.87\times Ratio^{2}-0.041\times DIM,$$

where *Ratio* = [MP supply (g/d) − urinary endogenous MP loss (g/d)]/DE intake (digestible energy; MJ/d).

The MPY (g/d) using this predicted efficiency (**MPY_eff**) was calculated as follows:

[3] $$\begin{array}{l} MPY\_eff\;=\\ \left(\left(MPsup-TPuri\right)-\frac{TPfecal+TPscurf+TPgrowth}{Eff}\right)\times \frac{Eff}{100}\;, \end{array}$$

where *MPsup* = MP supply (g/d); *TPuri* = urinary endogenous losses, as protein (g/d); *TPfecal* = true protein metabolic fecal loss (g/d); *TPscurf* = true protein scurf exportation (g/d); *TPgrowth* = true protein growth deposition (g/d); and *Eff* = efficiency predicted as described in Equation 2.

All supplies and outputs were as estimated by NASEM (2021).

The 2 approaches for MPY predictions were applied to the 2 estimations of DMI by NASEM (2021), one based only on animal characteristics (**DMI_Ao_**; Equation 2–1 of the NASEM, 2021 book) and the other based on both animal and fiber characteristics of the ration (**DMI_A&R_**; Equation 2–2 of NASEM, 2021 book), resulting in 4 MPY predictions.

To assess the performances of MPY predictions, 3 main metrics were used: the concordance correlation coefficient (**CCC**), as described by Lin (1989), and the root mean square error (**RMSE**), as described by Bibby and Toutenburg (1977), which includes normalized RMSE (**NRMSE**), calculated as follows:

[4] $$RMSE=\frac{\sqrt{\sum_{i=1}^{n}{\left(obs_{i}-pred_{i}\right)}^{2}}}{n},$$

[5] $$NRMSE=\frac{RMSE}{obs_{mean}},$$

where *NRMSE* = normalized root mean square, *obs_i_* = *i*th observed value (g/d), *pred_i_* = *i*th predicted value (g/d), and *obs_mean_* = observed mean (g/d).

To assess accuracy and precision, the bias correction factor (**Cb**) and Pearson correlation (**r**) were used. Lin's CCC and Cb were evaluated using the *epi.ccc* function from the epiR package (Stevenson and Sergeant, 2021). Central tendency bias (**CTB**), regression bias (**RB**), and disturbance bias (**DB**), as described by Bibby and Toutenburg (1977), were also calculated to evaluate the type of error present in predictions. For CCC, Cb, and r, values closest to 1 are more desirable, whereas the smallest values of NRMSE, CTB, and RB are preferable.

Diet and cow characteristics are detailed in Table 1. As described in Binggeli et al. (2022, 2023), all cows were Holstein. Estimations of MP and AA supplies, and MP expenditures are detailed in Table 2. To stay consistent with the previous study, NASEM DMI estimation was used. The average DMI_Ao_ (23.4 kg/d) was higher than the average DMI_A&R_ (20.9 kg/d), and predicted MP supplies varied accordingly. Calculations for scurf, urinary endogenous, growth, and milk expenditures were not affected by the nature of DMI estimation, whereas the calculation for metabolic fecal MP expenditures, based on DMI, averaged 421 and 365 g/d for DMI_Ao_ and DMI_A&R_, respectively. Predicted efficiencies of utilization of MP (Equation 2) averaged 70.2% and 69.6%, respectively. Comparing both DMI highlights the effect of DMI change on total supply and nutrient concentrations as actual individual DMI was unknown.

**Table 2:** Predictions of DMI, MP supply and expenditures, and AA supply with the 2 DMI predictions from NASEM^1^

Item	DMI_Ao_		DMI_A&R_	
	Mean	SD	Mean	SD
Predicted DMI, kg/d	23.4	2.5	20.9	2.8
Predicted MP supply, g/d	2,059	279	1,853	277
From MCP^2^	1,257	114	1,145	121
From RUP	802	207	708	192
His supply, g/d	46	7	41	7
Ile supply, g/d	123	15	110	15
Leu supply, g/d	183	31	165	30
Lys supply, g/d	154	17	139	18
Met supply, g/d	45	6	41	6
Other AA,^3^ g/d	1,816	247	1,631	245
Σ(EAA^2^),^4^ g/d	133,934	35,028	109,749	31,560
His supply, % of MP	2.22	0.06	2.23	0.06
Lys supply, % of MP	7.53	0.34	7.58	0.34
Met supply, % of MP	2.21	0.04	2.22	0.04
DE intake, Mcal/d	71.5	8.1	62.8	8.8
DE intake (nonprotein), Mcal/d	56.4	6.2	49.6	6.7
DNDF,^5^ kg/d	5.7	0.9	4.8	0.7
Predicted MP expenditure,^6^ g/d	1,966	392	1,911	401
Maintenance	656	71	601	59
Scurf	12	1	12	1
Urinary endogenous	223	19	224	19
Metabolic fecal	421	57	365	54
Growth including mature cow	15	12	15	12
Growth without mature cows	26	1	26	1
Milk protein, g/d	1,295	250	1,295	250
MP efficiency calculated from Lapierre et al. (2023), %	70.2	6.6	69.6	6.8

1 DMI_Ao_ = DMI based on animal characteristics only (Equation 2–1; NASEM, 2021); DMI_A&R_ = DMI based on animal and ration characteristics (Equation 2–2; NASEM, 2021).

2 MCP = microbial crude protein.

3 NEAA + Arg + Phe + Thr + Trp + Val (Equation 20–186a; NASEM, 2021).

4 Sum of EAA squared; His^2^ + Ile^2^ + Leu^2^ + Lys^2^ + Met^2^ (Equation 20–186b; NASEM, 2021).

5 DNDF = digestible NDF.

6 All expenditures are based using the 69% target efficiency of utilization proposed by NASEM.

For both MPY_multi and MPY_eff prediction methods, DMI_A&R_ presented a better fit than DMI_Ao_ (Figure 1) with higher CCC and lower RMSE, CTB, and RB. As also observed in Figure 1, MPY_multi predictions were subject to a higher RB than MPY_eff with the 2 estimations of DMI. However, MPY_eff were subject to a higher proportion of error on the CTB than MPY_multi when DMI_Ao_ was used.

**Figure 1:**
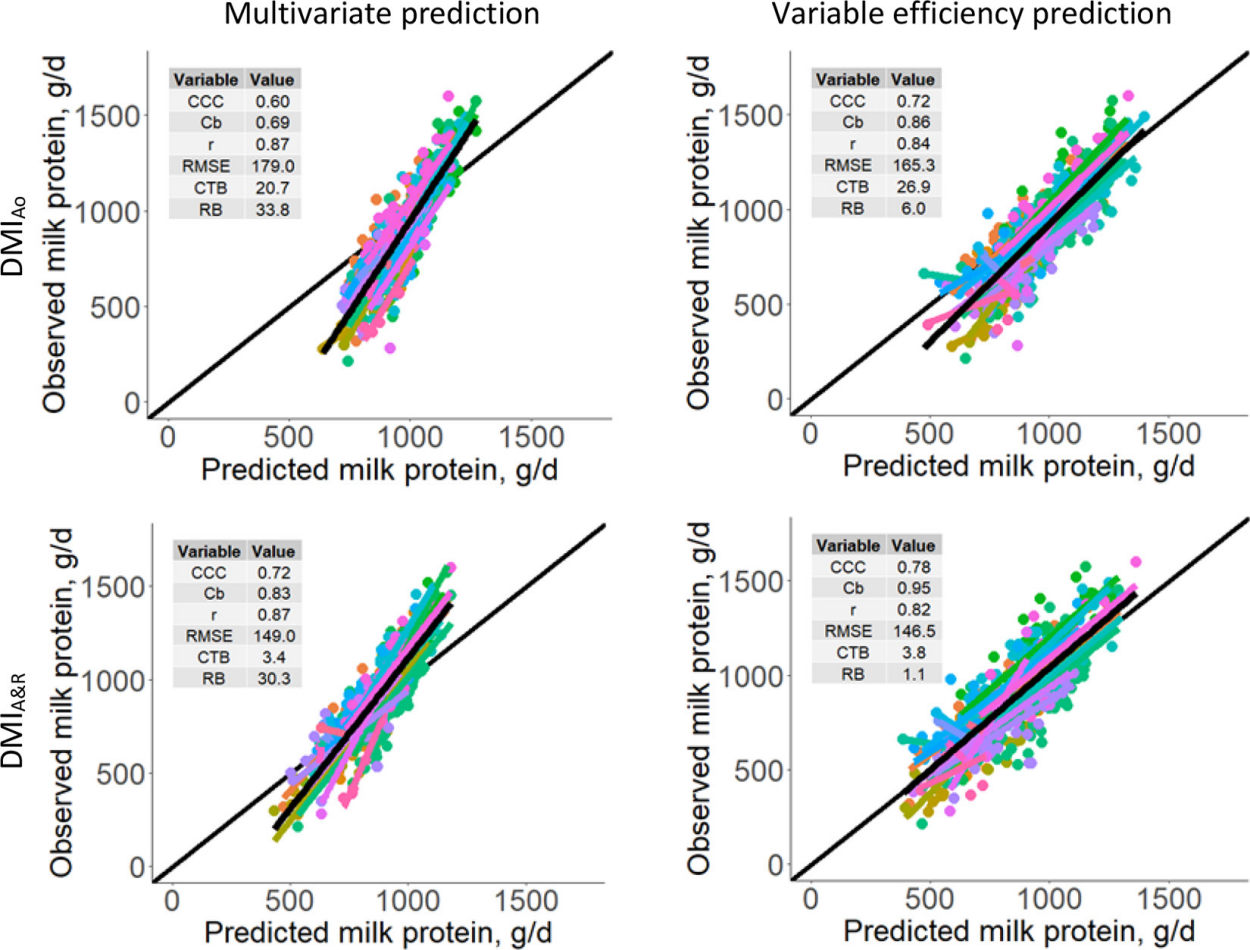
Relationship between observed and predicted milk protein yield using the 2 DMI predictions from NASEM (DMI_Ao_ = DMI based on animal characteristics only; DMI_A&R_ = DMI based on animal and ration characteristics). Each point represents a cow, each color a herd, the thin black line represents the bisector, and the thick black line is the general regression. CCC = concordance correlation coefficient (Lin, 1989); Cb = bias correction factor (Lin, 1989); RMSE = root mean square error; CTB = central tendency bias; RB = regression bias.

In its internal evaluation of the prediction of MPY using the multivariate equation, NASEM (2021; Table 6–3) had slightly better CCC (0.75) and NRMSE (14.4% of observed mean) than in the current study; the RB of only 3.1% of mean square error (**MSE**) in NASEM may explain these observations.

As mentioned before, Binggeli et al. (2022) compared 4 FEM using the same dataset and methodology. The NorFor system yielded the best MPY predictions with a CCC of 0.82 and an RMSE of 136.1 g/d. From the current study, using estimated DMI_A&R_, based on RMSE_,_ MPY_eff and MPY_multi would rank below NorFor and just before CNCPS. However, based on CCC, MPY_eff would rank below NorFor, whereas MPY_multi would rank last, below CNCPS, NRC, and INRA. The strong RB but small overall noise observed on Figure 1 would explain this discrepancy on performance evaluation for the MPY_multi. Using estimated DMI_Ao_ yielded the lowest CCC among all FEM.

The average MP concentration in NASEM (88 to 89 g of MP/kg of DMI) was slightly above the values reported for INRA (87 g of MP/kg of DMI), but below the values reported for NRC, NorFor, and CNCPS, averaging 90, 94, and 99 g of MP/kg of DMI, respectively (Binggeli et al., 2022). Accordingly, only INRA and NASEM do not include the contribution of the endogenous secretions to the MP supply. Among factors that may explain the variation in MP concentration between FEM, flows of RUP and microbial crude protein (**MCP**) appeared important. The NASEM RUP and MCP concentration predictions were intermediate between the values of the 4 other FEM, at 34 and 57 g/kg of DMI, respectively. It could be criticized that NASEM decided to implement fixed passage rates (**Kp**) of forage and concentrate to predict RUP. This contradicts the well-established principle of variability of Kp from diet composition (Seo et al., 2006; Krizsan et al., 2010), which is used in most FEM (i.e., NRC, CNCPS, NorFor, and INRA). However, the coefficient of variation of RUP for NASEM was similar to the other FEM (26% and 27% vs. 27% to 33%, for NASEM estimations vs. other models). This indicates that the fixed Kp seems somewhat acceptable, as variation is similar to models using variable Kp.

As observed on Figure 1, the MPY_multi tended to overestimate MPY at low production and underestimate at a higher production level. To explain why such a RB was not observed with MPY_eff, it was observed that residuals of MPY_multi were negatively correlated with DIM (r = −0.51 and −0.26 for DMI_Ao_ and DMI_A&R_, respectively, *P* ≤ 0.001). This may imply a reduction in efficiency of utilization as lactation progresses, as described by the variable efficiency from Lapierre et al. (2023), a factor not considered by the multivariate equation. Using the same commercial farms, this regression bias was also not observed with any of the 4 FEM studied, with all RB being lower than 3.5% of the MSE (Binggeli et al., 2022). However, the same DIM effect was also observed for the 4 other models, although to a lesser extent (Binggeli et al., 2022). NASEM (2021) is also one of the few FEM, with INRA, to consider a possible effect of genetic potential of animals on MPY predictions, via the 305-d milk true protein RHA. However, its effect seems negligible, as a ±15% variation of the RHA caused less than 1 point in percentage on the NRMSE, affecting slightly both the slope and intercept error (data not shown). Using the average herd RHA instead of individual RHA for each cow led to lower performances in all cases (data not shown). In addition, the residuals were positively correlated with the individual RHA (r = 0.13 and 0.30 for DMI_Ao_ and DMI_A&R_, respectively; *P* ≤ 0.005), which indicates an inaccuracy in the adjustment of the coefficients in the NASEM multivariate equation used to predict MPY response. The 15% increase in RHA decreased the slope of the relationship between new residuals and the RHA, but the correlation remained similar, for both DMI predictions. This is also in agreement with previous observations reporting that animals with higher genetic merit are potentially more efficient in protein utilization (Richardson and Van Doormaal, 2021; Binggeli et al., 2022). In NASEM, the RHA have been constructed to have the same curvature of response for different levels of potential, which contradicts the possible gain in efficiency for cows of different physiological status, genetic potential, or both. It is, however, mentioned in NASEM that a scalar can be used to correct this effect, but no suggestion has yet been made on how to implement it.

When NASEM is applied on commercial farms, the best predictions of MPY were obtained using DMI predicted with animal and ration characteristics paired with a variable efficiency of use of MP for MPY. The MPY predictions using the multivariate NASEM equation had a slope bias leading to underestimation of MPY at higher production level. The DMI predicted using only animal characteristics was associated with overpredictions of MPY. Nevertheless, the use of NASEM paired with a variable efficiency of utilization of MP holds promise for predicting MPY in commercial herds.
